# Effects of Polychlorinated Dibenzo-p-dioxins, Polychlorinated Dibenzofurans, and Dioxin-like PCBs on Teeth and Bones in Animals and Humans

**DOI:** 10.3390/toxics11010007

**Published:** 2022-12-21

**Authors:** Tomoya Takiguchi, Hoa Thi Vu, Yoshikazu Nishino

**Affiliations:** Department of Public Health, Kanazawa Medical University, Uchinada 920-0293, Japan

**Keywords:** dioxins, dioxin-like PCB, animals, human, teeth, bone

## Abstract

Bone metabolism is regulated by endocrine systems, so people exposed to polychlorinated dibenzo-p-dioxins and dibenzofurans (PCDD/Fs) may suffer adverse effects on bones and teeth. We reviewed previous publications in which effects of PCDD/Fs and dioxin-like polychlorinated biphenyls on the teeth and bones of animals and humans were found. The aim was to identify future research directions, particularly for epidemiological studies of populations exposed to PCDD/Fs in the environment. Exposure of fetuses to PCDD/Fs may affect odontogenesis, particularly enamel formation, but the effects of PCDD/Fs on bone genesis are limited to palatine bone. Exposure to PCDD/Fs in milk may affect both teeth and bones, but the effects on bones may be reversible. Exposure to high PCDD/F concentrations even during adulthood may adversely affect teeth. Exposure to PCDD/Fs may induce osteogenesis and improve bone properties because the disrupting effects of PCDD/Fs cause bone remodeling and vitamin D activation. More studies involving humans are required to investigate previously found associations between the PCDD/F concentrations humans are exposed to and biological markers for teeth and bones, including metabolites of vitamin D.

## 1. Introduction

There are 75 polychlorinated dibenzo-p-dioxin (PCDD) congeners and 135 polychlorinated dibenzofuran (PCDF) congeners. Together, PCDDs and PCDFs are often abbreviated as PCDD/Fs. Each PCDD congener has between one and eight chlorine atoms attached to a dibenzo-p-dioxin structure, and each PCDF congener has between one and eight chlorine atoms attached to a dibenzofuran structure. However, only congeners with chlorine atoms in the 2, 3, 7, and 8 positions are extremely potent/toxic and highly persistent to humans and most animals. There are seven PCDD congeners and 10 PCDF congeners with chlorine atoms at the 2, 3, 7, and 8 positions. These 17 PCDD/F congeners should therefore be determined in samples from humans. There are 209 polychlorinated biphenyl (PCB) congeners, but only mono-ortho-chlorinated biphenyls and non-ortho-chlorinated biphenyls have similar types of toxicities to PCDD/Fs. These PCBs are called dioxin-like (dl) PCBs. Non-ortho-chlorinated biphenyls are more toxic than mono-ortho-chlorinated biphenyls. Only four non-ortho-chlorinated biphenyl congeners have therefore been determined in some studies.

2,3,7,8-Tetrachlorodibenzo-p-dioxin (TCDD) is the most toxic PCDD/F congener and is a by-product of the production of herbicides, such as 2,4-dichloroacetophenol and 2,4,5-trichloroacetophenol. TCDD causes chloracne in occupationally exposed people. Suskind and Hertzberg (1984) performed a clinical epidemiological study aimed at identifying the health effects caused by industrial exposure of workers involved in manufacturing 2,4,5-trichloroacetophenol (which was contaminated with TCDD) [1]. They found a higher prevalence of chloracne (55.7%) associated with actinic elastosis of the skin in 204 exposed workers than in non-exposed workers [1].

A three-year-old girl was found to have chloracne in 1968, and many people in southern parts of Japan were subsequently found to have Yusho disease, which was caused by the consumption of rice oil contaminated with PCBs and PCDFs. Yamashita and Hayashi (1985) found that infants exposed to PCBs as fetuses had peculiar clinical symptoms including cola-colored skin, gingival hyperplasia, natal teeth, a large fontanelle, and unusual calcification of the skull bone (fetal PCB syndrome) [2]. In the same parts of Japan, lower body weights and heights were found for girls without Yusho symptoms but with mothers with breast milk containing high PCB concentrations compared to unexposed girls [2]. This indicated that organochlorine compounds, such as PCBs and PCDD/Fs, may alter calcium metabolism through endocrine disruption and cause a decrease in body size.

In the 1990s, the effects of PCDD/Fs on teeth in animals and humans were investigated in Finland, and developmental enamel defects were found to be the most sensitive effects of PCDD/Fs exposure ever known, and found to be important and useful markers of exposure to PCDD/Fs and dioxin-like compounds in humans [3,4]. However, very few epidemiological research works have investigated the effects of PCDD/Fs on teeth using markers of developing teeth [5,6].

Bone metabolism is regulated by endocrine systems, particularly estrogen signaling, so PCDD/Fs were expected to adversely affect bones in animals and humans. However, PCDD/Fs have not been found to adversely affect bones (e.g., by decreasing bone mass or increasing osteoporosis occurrence) in people exposed to high concentrations of PCDD/Fs and dl-PCBs, such as women accidentally exposed to high TCDD concentrations in Seveso, Italy [7].

In this review, we summarize previous reports of the effects of PCDD/Fs and dl-PCBs on teeth and bones in animals and humans and assess the differences between the results to allow future directions for studies of populations exposed to PCDD/Fs to be suggested.

## 2. Materials and Methods

Eligible studies were identified by searching the literature using Pub Med in English. The search terms were “dioxins” OR “TCDD” OR “dioxin like PCBs” AND “bone” OR “teeth”. Only original articles were selected, and the studies described in the articles were divided into two groups after excluding in vitro studies: in vivo animal studies and human studies (including epidemiological and clinical studies) of teeth and bones. The animal and human studies were divided into two groups based on the exposure period: the first group was exposure during the fetal and lactational period, and the second group was exposure during adulthood including the juvenile period. This was because the sensitivity of animals and humans to toxicants’ exposure is highly dependent on the stage of development.

## 3. Results

### 3.1. Effects of PCDD/Fs and dl-PCBs on Tooth Growth and Oral Health

#### 3.1.1. Animal Studies

The effects of gestational and lactational exposure to TCDD on tooth growth and the effects of exposure to high TCDD concentrations in adulthood on tooth growth were investigated in some animal studies aimed at investigating the adverse health effects associated with exposure to PCDD/Fs (Table 1).

##### Effects of Gestational and Lactational Exposure to TCDD on Tooth Growth

Kattainen et al. (2001) investigated the effects of gestational and lactational exposure to low maternal TCDD doses (0.03–1.0 µg/kg) on tooth development (mainly third molars, which develop from the perinatal period to about six weeks old) in three strains of rats with different sensitivities to TCDD and different aryl hydrocarbon receptor (AhR) structures [8]. The third molar eruption inhibition rate caused by exposure to TCDD at the same dose was much higher in pups of the most susceptible strain than pups of the other strains [8]. TCDD at 1 mg/kg completely prevented the development of the third lower molars in 60% of males and 50% of females in the most sensitive rat line. The percentage of third molars that had erupted in five-week-old pups of the most susceptible strain decreased as the TCDD dose increased. The sizes of the erupted molars in pups of all of the strains (i.e., regardless of AhR sensitivity) decreased as the TCDD dose increased [8].

Lukinmaa et al. (2001) administered TCDD (at a dose of 50 or 1000 µg/kg) to PCDD/F-resistant Han/Wistar (H/W) rats on postnatal day 1 and investigated the radiographical and histological differences in tooth development in the pups on postnatal days 9 and 22 [9]. Third molars were more frequently missing and more molars in the maxilla were missing in the high-TCDD-dose (1000 µg/kg) group than the low-TCDD-dose group (50 µg/kg) [9]. Eruption of the third molars was delayed and the erupted third molars were not calcified (determined by radiographic examination) in both the high- and low-TCDD-dose groups [9]. First and second molar root formation ceased early but eruption was not affected. Dentin formation in the erupted mandibular incisors stopped at the pre-eruption stage because of pulp cell death [9]. These results suggest that the first, second, and third molars and the incisors are affected by lactational exposure to TCDD even in strains less sensitive to TCDD/AhR.

Miettinen et al. (2002) determined the critical window during gestational and lactational exposure to TCDD for effects on development of molars in rat pups using a maternal TCDD dose of 1 µg/kg [10]. The pups exposed to TCDD on days 11, 13, and 19 of gestation had missing third molars, and earlier lower incisor eruption was found at 2 days old, indicating that TCDD accelerated incisor eruption but delayed third molar eruption [10]. The most sensitive period for effects on third molar development was the early tooth formation stage (11–19 days of gestation) [10].

Gao et al. (2004) administered TCDD (at a dose of 50 or 1000 µg/kg) to H/W rats the day after birth and found enamel matrix stagnation (retention) and thicker predentin in the upper first and second molars of the exposed rats (at both doses) than of control rats [11]. Observations of immune-stained ameloblasts and odontoblasts suggested that TCDD inhibited tooth calcification by decreasing CYP1A1 activity via the AhR [11].

Thus, gestational and lactational exposure to TCDD affects the development of molars in rats, resulting in defects, delayed eruption, and impaired calcification. It has been found that the most sensitive period to TCDD exposure is the early stage of tooth morphogenesis in utero and that the mechanisms involved include ameloblast and odontoblast inhibition preventing calcification of the teeth via the AhR.

##### Effects of Chronic Exposure to TCDD at High Doses in Adulthood on Teeth

Alaluusua et al. (1993) examined the continually erupting portal teeth of young male rats 16 weeks after administering a single TCDD dose at 1000 μg/kg [12]. The upper and lower incisors were markedly thinner in the exposed group than the control group, and the pulp in the lower incisors in the exposed group was lingually exposed to the oral cavity at the incisor ends [12]. The labial surfaces of the incisors were grayish and mottled in the exposed group but brown in the control group [12]. Histological examination indicated that the pulp chambers in the affected incisors were larger (at the expense of dentin) in the exposed group than the control group [12]. The odontoblasts on the incisal sides gradually lost polarity and the pulp tissue became necrotic in the exposed group, and the dentin next to the pulp chamber was irregular [12]. The roots in the exposed group were markedly tapered and had mesiodistally flattened appearances. The superficial enamel layers in the exposed group were pigmented [12]. These results indicated that chronic exposure to TCDD affects all tissues of the incisors in young male rats.

Kiukkonen et al. (2002) exposed TCDD-resistant H/W rats and susceptible female Long–Evans (L/E) rats to TCDD at total doses of 0.17, 1.7, 17, and 170 µg/kg given as weekly doses for 20 weeks from 10 weeks of age and observed the effects on the incisors [13]. Exposure of both strains to TCDD doses of 17 and 170 µg/kg resulted in color defects and pulpal perforation in the lower incisors, and periodontal ligament and pulp cell necrosis and consequent arrest of dentin formation at the incisal ends were found during histological examination [13]. The incisors of H/W rats were more affected, resulting in larger perforations at TCDD doses ≥17 µg/kg than at TCDD doses < 17 µg/kg. The enamel-producing cells underwent early squamous epithelialization and marked proliferation accompanied by enamel discoloration [13]. These results suggested that relatively high TCDD doses affected both the mesenchymal and epithelial elements of the forming tooth. However, incisor tooth formation impairment was not markedly different for the rat strains with different AhR sensitivities [13].

Thus, exposure to TCDD at relatively high doses in adulthood affects both the mesenchymal and epithelial elements (the two elements involved in tooth formation) of the incisors, resulting in dentin formation ceasing and qualitative changes in the enamel. However, pathways other than AhR signaling may mediate the effects of TCDD on teeth in adult rats.

#### 3.1.2. Studies of Humans: Epidemiological Studies

In some studies, natal teeth were found at birth in infants with fetal PCB syndrome (called Yusho disease) in Japan [2,14]. Infants with fetal PCB syndrome (called Yu-cheng disease) in Taiwan had similar symptoms to infants with Yusho disease in Japan, and natal teeth were found at birth [15,16]. However, no symptoms involving teeth were found in follow-up studies of children with Yu-cheng disease [17]. No greater number of natal and neonatal teeth were found in children exposed to PCBs than in non-exposed children in Finland [18]. Adverse effects on teeth other than natal teeth in patients with Yusho or Yu-cheng disease are therefore described below (Table 2).

##### Fetal and Lactational Exposure to PCDD/Fs

Alaluusua et al. (1996) investigated the association between exposure to PCDD/Fs in breast milk and enamel dysplasia in Finnish children aged 6–7 years and found that exposure to PCDD/Fs was associated with increased frequency and severity of enamel dysplasia in the permanent first molars mineralized during the first 2 years of life [4]. The association with enamel defects was stronger for PCDD/Fs than PCBs, suggesting that developing teeth might be good indicators of adverse health effects caused by exposure to PCDD/Fs early in life.

Laisi et al. (2008) investigated associations between the occurrence and severity of hypo-mineralization of molars and incisors and exposure to PCDD/Fs and PCBs (indicated by the PCDD/F and PCB concentrations in the placentas) for Finnish mother–infant pairs, but no associations were found [5].

Few epidemiological studies have been performed to investigate associations between fetal and/or lactational exposure to PCDD/Fs and growing teeth in infants. The effects of exposure of children to PCDD/Fs and dl-PCBs using a birth cohort with quantified perinatal exposure should be studied.

##### Exposure to PCDD/Fs in Childhood and Adulthood

Fukuyama et al. (1979) investigated tooth development in patients with Yusho disease [19]. Tooth eruption was delayed in 18% of patients with oiliness from 0 to 14 years of age [19]. Delayed tooth eruption was a standard symptom in the diagnostic criteria for Yusho disease [21]. Abnormal root morphology was found in 77% of patients aged 0 to 15 years [19]. These results suggested that exposure to PCBs may affect tooth development in children.

Guo et al. (1999) monitored patients with Yu-cheng disease and controls for 14 years after the disease outbreak and compared the prevalence of reported problems, including gum pigmentation and broken teeth, diagnosed in a clinic or hospital [20]. Broken teeth were around two times as prevalent in patients of both sexes than the controls, and the odds ratios for gum pigmentation were much higher for both sexes (5.6 for men and 8.5 for women) [20]. However, it was not clear why teeth were easily broken.

Alaluusua et al. (2004) investigated dental and oral abnormalities in an area contaminated because of an explosion at a pesticide plant in Seveso, Italy, and in a lightly contaminated area [6]. Of subjects with enamel dysplasia, 93% were <5 years old at the time of the accident [6]. In this age group, 42% of the subjects with defects lived in contaminated areas and 26% lived in lightly contaminated areas, and the defects were associated with the TCDD concentrations in serum [6]. Tooth defects were found in 12.5% of the subjects living in polluted areas and 4.6% of the subjects living in lightly polluted areas and were associated with the TCDD concentrations in serum [6].

Kanagawa et al. (2008) monitored Yusho patients for >30 years and investigated associations between symptoms including tooth pigmentation and the PCB, PCDF, and polychlorinated quarterphenyl (PCQ) concentrations in serum by performing logistic regression analysis after selecting symptoms by performing principal component analysis [21]. High PCQ concentrations in serum (≥0.10 µg/L) significantly increased the risk of tooth pigmentation, suggesting that exposure to PCQs may negatively affect teeth even a long time after exposure ceases [21].

Ngoc et al. (2019) performed a case–control study to investigate the effects of PCDD/Fs on the prevalence of enamel dysplasia in 2200 adults living in herbicide-sprayed and non-sprayed areas in Viet Nam [22]. The results indicated that enamel dysplasia occurred in 20.5% and 5.8% of adults living in the sprayed areas and the non-sprayed areas, respectively, suggesting that enamel dysplasia was almost twice as prevalent in the exposed population than in the control population [22]. Enamel dysplasia was more prevalent in the premolars than in the molars. Most lesions were found on the buccal surfaces of the teeth [22].

The results described above indicated that tooth loss, broken teeth, and enamel defects were more likely to occur in people exposed to high PCDD/F and PCB concentrations (e.g., Yusho patients and residents of Seveso, Italy) than in people not exposed to high PCDD/F and PCB concentrations. In the study performed in Viet Nam, only the prevalence of enamel dysplasia in exposed and unexposed populations was compared, and dose–response relationships between exposure to PCDD/Fs and tooth problems were not investigated. Studies of populations with quantified exposure to PCDD/Fs need to be performed to clarify the effects of exposure to PCDD/Fs on teeth and gums.

### 3.2. Effects of PCDD/Fs and dl-PCBs on Bone Growth and Remodeling

#### 3.2.1. Animal Studies

Associations between exposure to high TCDD doses and bone development during the perinatal or lactational periods and during adulthood have been investigated. Dental development primarily occurs during pre- and early postnatal periods, whereas bone development continues through adolescence, with maximum bone mass occurring in the juvenile period and bone remodeling continuing throughout adulthood. Morphological and biochemical effects on bone with chronic exposure to TCDD were therefore investigated in all periods of life (Table 3).

##### Effects of Fetal and Lactational Exposure to TCDD on Bone Growth

Miettinen et al. (2005) investigated the effects of fetal and lactational exposure to TCDD on the bones of three strains of rats with different AhR susceptibilities [23]. In the most susceptible strain, exposure to TCDD at a maternal dose of 1 µg/kg decreased bone length, cortical cross-sectional area, and bone density but did not decrease flexural fracture strength and tibia, femur, and femoral neck stiffness [23]. These effects were more severe in pups’ exposure earlier than later. No effects were found when only fetal exposure occurred, suggesting that lactational exposure was essential for bone impairment [23]. Most of the abnormalities were reversed in the first year of life, suggesting that most of the effects were reversible.

Nishimura et al. (2009) administered TCDD at a dose of 15 µg/kg orally to mouse dams on day 1 after delivery and found that the 1,25-dihydroxy vitamin D3 concentration doubled, and osteocalcin, collagen type 1, and alkaline phosphatase gene expression decreased markedly [24]. Abnormal calcification of the tibia, decreased osteoblastic osteogenic activity, and increased fibroblast growth factor 23 occurred in the exposed pups [24]. Through histomorphometry, it was found that TCDD altered osteoblastic activity but did not alter bone resorption activity [24]. The most prominent bone lesions were increased osteoid volume and thickness in the cortical and trabecular bones [24]. These results suggested that upregulation of vitamin D induced by exposure to TCDD decreases osteoblast activity, resulting in impaired bone mineralization.

Finnila et al. (2010) studied the effects of perinatal exposure to TCDD on bone matrix maturation in rats [25]. Pregnant rats received an intragastrical dose of 1 µg/kg of TCDD on gestation day 11, and the tibia properties of the offspring were assessed on postnatal days 35 and 70 [25]. Normal development, such as decreased plasticity and increased dynamic hardness, storage modulus, and composite modulus, occurred in the controls, and changes related to normal development did not occur in the offspring exposed to TCDD, indicating that bone matrix maturation was delayed [25]. Decreased bone calcification, shortened tibia length, and decreased strength were observed in the exposed pups [25]. These results suggested that TCDD-induced decreases in bone strength are associated with changes in bone mineralization and shape rather than changes in the bone matrix.

Yamada et al. (2014) administered TCDD at a dose of 40 µg/kg to mouse dams at 12.5 d of gestation and investigated the effects of exposure to TCDD on palatal development in the embryos at 15.5 d of gestation [26]. Immunoreactivity of Runx2 and osteopontin in the palatine bone and MyoD and desmin in the palatine muscle was lower in the embryos exposed to TCDD than in the control embryos [26]. Immunoreactivity of the AhR and ER-α was localized in the normal palatine bone, but ER-α activity in the palatine bone was decreased in the exposed embryos [26]. Western blot analysis indicated that Runx2, MyoD, and desmin in the palate were downregulated in the embryos exposed to TCDD [26]. These results suggested that TCDD may impair palatal osteogenesis and myogenesis via the AhR.

These results suggested that lactational exposure to TCDD affects bone growth primarily by affecting vitamin D and osteoblast activation, resulting in impaired bone mineralization and shape. Lactational exposure to TCDD may also negatively affect bone matrix maturation, resulting in altered bone material properties. Fetal exposure to TCDD impairs osteogenesis of the palatine bone, causing a cleft palate.

##### Effects of Exposure to TCDD in Adulthood on Bones

Alaluusua et al. (1993) administered a single dose of TCDD to young adult TCDD-resistant male H/W rats and found smaller skulls and thinner incisors than in the control rats 16 weeks after exposure [12]. They suggested that the impairment of skull and teeth formation may have been associated with exposure to TCDD affecting vitamin A metabolism [12].

Focusing on the sensitivity to the effects of TCDD being related to the AhR, Jamsa et al. (2001) investigated the effects of exposure to TCDD on bones in L/E rats that were very sensitive to exposure to TCDD and in H/W rats that were resistant to the effects of TCDD [27]. TCDD was administered to each rat once a week for 20 weeks starting at 10 weeks old. TCDD only affected the bones in H/W rats when the total TCDD dose was 170 µg/kg, but a marked decrease in bone growth (e.g., decreased tibia size) occurred in the L/E rats at a TCDD dose of 1.7 µg/kg [27]. However, bone density in the epiphyseal region remained unchanged in the L/E rats [27]. The breaking force of the tibia from the H/W rats was decreased at a TCDD dose of 170 µg/kg and stiffness of the tibia from the H/W rats was decreased at a TCDD dose of 17 µg/kg [27]. The breaking force and stiffness of the tibia from the L/E rats were both decreased at a TCDD dose of 17 µg/kg [27]. These results suggested that the AhR may be involved in the effects of TCDD on bone growth, modeling, and mechanical strength.

Herlin et al. (2010) percutaneously administered TCDD at doses of 1–1000 ng/kg/day to 10-week-old female L/E and H/W rats once each week for 20 weeks (0.14–140 µg/kg of total doses) and quantified changes in bone shape, calcification, and biomechanical properties caused by long-term exposure to TCDD [28]. The results indicated that TCDD affected the geometrical and biomechanical parameters of the bone. The strongest responses were found in the trabecular bone area of the proximal tibia and the intracortical circumference of the tibial epiphysis [28]. The most marked difference (by a factor of ~49) between the two rat strains was for the cross-sectional area of the proximal tibia, indicating that the AhR plays a role in bone toxicity caused by TCDD [28].

Herlin et al. (2013) administered TCDD weekly for 10 weeks at a total dose of 200 µg/kg to AhR knockout and wild-type mice and used molecular biological methods to confirm that the AhR was involved in bone toxicity caused by TCDD [29]. The bone matrices were stiffer, the cortical bones were thinner and more porous, and the trabecular bone compartments were more compact in the wild-type mice exposed to TCDD than in the AhR knockout mice exposed to TCDD [29]. Exposure to TCDD also affected the expression of bone metabolism markers and osteogenesis-related genes, suggesting that an imbalance in bone remodeling occurred. In contrast, exposure to TCDD caused minimal bone changes in the knockout mice [29]. These results suggested that the AhR is involved in normal bone development and plays an important role in the osteo-toxic mechanism of TCDD.

Fader et al. (2018) investigated the effects of TCDD (at doses of 0.01–30 µg/kg) on the femurs of juvenile mice and found dose-dependent increases in the trabecular bone volume in both sexes [30]. Exposure to TCDD decreased serum tartrate-resistant acid phosphatase levels and osteoclast surface-to-bone surface ratios and inhibited femoral bone proteases, such as cathepsin K and matrix metallopeptidase 13, indicating that TCDD inhibited bone resorption [30]. Exposure to TCDD also increased the number of osteoblasts and decreased the number of bone marrow adipocytes on the trabecular bone surface, suggesting that the AhR may activate the differentiation of mesenchymal stem cells into osteoblasts. Analysis using an RNA sequencer indicated that transmembrane glycoprotein NMB expression and expression of a positive regulator of osteoblast differentiation and mineralization were dose-dependently induced by exposure to TCDD [30]. Exposure to TCDD also increased the 1,25-dihydroxy vitamin D3 concentration in serum, consistent with induction of 1α-hydroxylase Cyp27b1 in the kidneys, indicating that TCDD may have contributed to impaired bone resorption [30]. These results suggested that AhR activation by TCDD may shift bone remodeling toward bone formation and decrease the production of bone marrow fat.

Thus, the exposure of juveniles to TCDD altered bone shape, decreased bone size, and decreased the bone breaking force and stiffness, particularly of the tibia, by affecting bone remodeling via the AhR. However, TCDD was not found to affect bone density. Inconsistent positive effects of TCDD on bone remodeling have also been reported. TCDD exposure has been found to increase bone formation in the femur by inhibiting bone resorption via vitamin D activation and increased activation of osteoblast differentiation from mesenchymal stem cells. Further studies will be required to clarify the effects of TCDD on various bones and at lower TCDD doses.

#### 3.2.2. Studies of Humans: Epidemiological and Clinical Studies

No epidemiological studies aimed at investigating associations between bone parameters and perinatal exposure to PCDD/Fs have been published. The human studies described here used populations mainly exposed to PCDD/Fs during childhood and adulthood (Table 4).

Akamine et al. (1985) found that alveolar bone resorption occurred in at least one tooth in 56.4% of Yusho patients and more commonly in men than women [31]. Severe alveolar bone resorption was found in patients in their 20 s and 30 s. Shimizu et al. (1992) monitored Yusho patients for 12 years and found that many patients had periodontal disease with horizontal alveolar bone resorption despite good oral care [32]. These clinical results suggested that mineralization of the alveolar bone may be affected by exposure to PCDD/Fs.

Hodgson et al. (2008) used dual-energy X-ray absorptiometry (DEXA) to measure the bone mineral densities (BMDs) of the forearm bones of 154 men and 167 women 60–81 years old living near a PCB-contaminated river on the Baltic Sea coast and also assessed the relationships between the BMDs and the concentrations of five dl-PCBs, three non-dl-PCBs, and p,p′-dichlorodiphenyldichloroethylene [33]. In men, the PCB-118 (a dl-PCB) concentration negatively correlated with the BMD, and the odds ratio was 1.06 (95% confidence intervals 1.01 and 1.12) for each increase of 10 pg/mL in the PCB-118 concentration for a low BMD (Z-score < −1) [33]. However, the sum of the concentrations of the three most abundant non-dl-PCB positively correlated with the BMD [33]. In women, the PCB-118 concentration positively correlated with the BMD and exposure to PCBs was not found to be a risk factor for decreased BMD [33].

Eskenazi et al. (2014) investigated the association between exposure to TCDD in youth and the bone structure and skeleton in adulthood [7]. They also investigated whether the timing of exposure to TCDD affected the association [7]. DEXA bone density measurements of the lumbar spine and hip and hip structural analysis measurements to determine size and strength indices for the three hip-forming bone regions were performed on 350 women in Seveso, Italy, who were <20 years old at the time of the Seveso accident [7]. The relationships between these bone indices and the TCDD concentrations in serum samples taken from the women immediately after the pesticide plant explosion were assessed [7]. The results indicated that the TCDD concentration was associated with several indicators of good bone structure in premenopausal women exposed to TCDD before peak bone mass had been reached [7]. The association was stronger for women exposed to TCDD before the age of 5 years old. Better bone structure was found for postmenopausal women exposed to TCDD after peak bone mass had been achieved than was found for postmenopausal women exposed to TCDD before peak bone mass was achieved [7]. These results suggested that exposure to TCDD may strengthen bones and not negatively affect bones in women before or after menopause.

Fukushi et al. (2016) assessed associations between PCDD, PCDF, and non-ortho-chlorinated biphenyl concentrations in serum and BMD determined by dual-energy X-ray absorptiometry in 262 female and 227 male Yusho patients [34]. After adjusting for area differences, the 1,2,3,4,7,8-hexachlorodibenzo-p-dioxin and 2,3,7,8-tetradibenzofuran concentrations were found to significantly positively correlate with the BMD Z-score for males [34]. No significant correlations were found between the PCDD, PCDF, and non-ortho-chlorinated biphenyl concentrations in serum and the BMDs for females. However, after adjusting for area and body mass index, the 1,2,3,4,6,7,8-heptachlorodibenzo-p-dioxin concentration was found to negatively correlate with the BMD Z-score for females [34]. These results indicated that exposure to some PCDD/F congeners may decrease bone density in women. Studies with more confounding factors controlled will be required to determine more clearly the effects of PCDD/Fs on bone density in Yusho patients.

No clear adverse effects of PCDD/Fs on bone density leading to osteoporosis were found in the epidemiological studies mentioned above, although altered mineralization of alveolar bone may have occurred in people exposed to high PCDD/F concentrations (e.g., Yusho patients). Better bone structure was associated with exposure to TCDD in women affected by the Seveso accident, but this may have been caused by TCDD causing bone remodeling to occur as bone formation, as was found in an animal study performed by Fader et al. (2018) [30].

## 4. Discussion

### 4.1. Effects of PCDD/Fs on Teeth and Their Potential Mechanisms

In animals, fetal and lactational exposure to TCDD affect the development of molars, resulting in defects, delayed eruption, and impaired calcification. The early stages of tooth morphogenesis in the fetal period are most sensitive to TCDD. In vitro studies of TCDD effects on the process of tooth formation using mouse molar embryos, it was reported that the impairment of epidermal growth factor receptor (EGFR) [35] and promoting epithelial apoptosis [36] may play important roles in the toxic effects of TCDD in fetuses. Moreover, Kiukkonen et al. (2006) investigated the associations between impaired mineralization of mandibular molars in mouse embryo budding stage of E18 by TCDD and expression of dentin sialophosphoprotein (Dspp), Bono1, and matrix metalloproteinase-20 (MMP-20), which are involved in hard tissue calcification [37]. Although no clear differences in the localization and intensity of Bono1 and MMP-20 expression were observed, Dspp expression in secretory odontoblasts and in presecretory ameloblasts was reduced in embryos incubated with TCDD. These findings suggest that TCDD may cause impaired calcification of dentin due to toxicity specific to Dspp expression.

Exposure to relatively high TCDD doses in adulthood can affect both mesenchymal and epithelial elements in the incisors, resulting in cessation of dentin formation and qualitative changes in the enamel [13]. However, no difference of impaired formation of the incisor tooth was found between rat strains with different AhR sensitivities [13], suggesting a pathway other than AhR signaling might mediate the effects of high TCDD exposure on teeth in adult rats.

In human studies, delayed tooth eruption and broken teeth occurred in Yusho patients exposed to high PCB, PCQ, and PCDF concentrations. Enamel dysplasia has been suggested to be a characteristic symptom in Finnish children exposed to high PCDD/F concentrations, and residents of Seveso, Italy, exposed to extremely high TCDD concentrations. However, associations between PCDD/F concentrations and tooth growth, including enamel dysplasia, have been investigated only in three studies [4,5,6] with populations whose perinatal exposure to PCDD/Fs was quantified, one of which showed no association between PCDD/Fs and growth of molars. More studies of perinatally exposed populations will be required to clarify the effects of exposure to PCDD/Fs on teeth.

### 4.2. Effects of PCDD/Fs on Bone and Their Potential Mechanisms

In animal studies, lactational exposure to TCDD was found to affect vitamin D and osteoblast activation in the tibia [24], resulting in impaired bone mineralization and shape and altered bone material properties. Fetal exposure to TCDD impaired osteogenesis and myogenesis of the palatine by decreasing Runx2 and osteopontin in the palatine bone and MyoD and desmin in the palatine muscle, leading to cleft palates [26].

In juvenile rats exposed to TCDD, the tibia shape was altered, and the size, breaking force, and stiffness were decreased but no effect of TCDD on bone density was found. However, exposure to TCDD at low doses increased bone formation in femurs by inhibiting bone resorption via vitamin D activation and increased activation of osteoblast differentiation from mesenchymal stem cells [30].

In an in vitro study using a system in which stem cells were isolated from the bone marrow of rat and mouse femurs and tibias, Korkalainen et al. (2009) investigated the effects of TCDD exposure including low dose of TCDD, e.g., 100 fM, on differentiation of osteoblasts and osteoclasts [38]. During osteoblast differentiation, TCDD significantly and dose-dependently decreased mRNA levels of RUNX2, alkaline phosphatase, and osteocalcin. In the case of osteoclasts, TCDD also reduced the number of TRACP+ multinucleated cells, with a concomitant decrease in the number and resorption area of F-actin rings. Effects on osteoblasts and osteoclasts occurred at very low doses of TCDD exposure. Taken together, the disturbance of osteoblast differentiation may also affect osteoclast formation resulting in the qualitative changes in bone caused by TCDD observed in in vivo studies.

Liu X et al. (2020) firstly confirmed that TCDD decreased cell proliferation and calcium deposition of the osteoblasts from human fetal palatal mesenchymal cells [39]. Then, they investigated molecular mechanisms of TCDD-induced inhibition of these osteogenic cells and reported that osteogenic cell differentiation was inhibited by downregulation of BMP-2/TGF-β1/Smad pathway via AhR signaling, suggesting that crosstalk between AhR and BMP-2/TGF-β1/Smad signaling may have an important role in osteogenic impairment of fetal palatal bone by TCDD [39].

In humans, no clear adverse effects of PCDD/Fs on bone were found in epidemiological studies using bone density as an indicator of osteoporosis. Better bone structures were associated with exposure to TCDD in women exposed to high TCDD concentrations in Seveso, Italy. However, this may have been caused by TCDD causing bone remodeling to occur in the direction of bone formation, as was found in an animal study.

## 5. Conclusions

Fetal exposure to PCDD/Fs appears to alter odontogenesis, particularly enamel formation, but has a limited effect on only palatine bone genesis. Lactational exposure to PCDD/Fs may affect both teeth and bones, but the effects on bones may be reversible. Exposure to high PCDD/F concentrations may affect teeth even during adulthood, but exposure to PCDD/Fs may induce osteogenesis and improve bone properties because PCDD/Fs may disrupt bone remodeling. Contrary to bones, teeth, and particularly the enamel of teeth, are not remodeled after birth, suggesting that developmental enamel defects can be used as biomarkers of PCDD/Fs exposure.

More epidemiological studies of humans are required to clarify associations between exposure to PCDD/Fs and biological markers of teeth and bones (including vitamin D metabolites) in the future.

## Figures and Tables

**Table 1:** Effects of TCDD on teeth in animals.

No.	Authors	Publication Year	Animals	Exposure Time	Exposure Dose of TCDD	Health Effects (Outcomes)
Exposure during fetal period and/or lactation						
1	Kattainen et al. [8]	2001	Han/Wistar (H/W) and Long-Evans (L/E) rats	Gestation day (GD) 15	0.03–1 μg/kg of a single oral dose	Lower percentage of erupted third molars (60% in males and 50% in females exposed to TCDD at 1 mg/kg) and smaller molar size
2	Lukinmaa et al. [9]	2001	H/W rats	1 day after delivery	50 or 1000 μg/kg	Abnormal development of molars and arrested dentin formation in lower incisors
3	Miettinen et al. [10]	2002	Dioxin-sensitive rats	GD11, GD13, GD19, postnatal day (PND)0, PND2, PND4	1 μg/kg	Accelerated eruption of the lower incisors and retarded eruption of the third molars.
4	Gao et al. [11]	2004	H/W rats	The day after delivery	50 or 1000 μg/kg	Enamel matrix stagnation and thicker predentin in the first and second molars
Exposure during adulthood						
1	Alaluusua et al. [12]	1993	TCDD-resistant H/W young adult male rats	11–12 weeks of age	1000 μg/kg of a single intra peritoneal dose	Thinner upper and lower incisors 16 weeks after exposure
2	Kiukkonen et al. [13]	2002	Resistant H/W female rats and susceptible L/E female rats	10–30 weeks of age	0. 17, 1.7, 17, 170 (H/W rats only) μg/kg	Mesenchymal and epithelial elements formation of incisors formation

H/W: Han/Wistar; L/E: Long/Evans; GD: Gestational day; PND: Postnatal day.

**Table 2:** Effects of dioxins and PCBs on teeth in humans.

No.	Author	Publication Year	Country/Region	Target Population (Gender, Age, Residents/Patients)	Exposure Indicators	Health Effects (Outcomes)
Dioxin exposure during fetal and/or lactation period						
1	Alaluusua et al. [4]	1996	Finland	Children aged 6–7 years (residents)	PCDD/Fs in maternal breast milk	Enamel dysplasia of permanent first molars.
2	Laisi et al. [5]	2008	Finland	Mothers and their 167 children	PCDD/Fs and PCBs in placentas	No effects on the child’s molars.
Dioxin exposure during childhood and adulthood						
1	Fukuyama et al. [19]	1979	Japan	Patients with Yusho (oil disease in Japan) aged at 0 to 15 years	Patients diagnosed as Yusho disease	Delayed eruption of permanent teeth, abnormal number of teeth, and abnormal root shape.
2	Guo et al. [20]	1999	Taiwan	Patients with Yu-cheng (oil disease in Taiwan)	Patients and controls	High prevalence of broken teeth (reported clinical history) 14 years after of diagnosis.
3	Alaluusua et al. [6]	2004	Seveso, Italy	Residents exposed to TCDD from industrial explosion	Serum TCDD concentration soon after accident	Enamel dysplasia and defects of teeth.
4	Kanagawa et al. [21]	2008	Japan	Patients with Yusho (oil disease in Japan)	Serum levels of PCBs, PCDFs, and polyquarterphenyls (PCQs)	Tooth pigmentation 30 years after of diagnosis.
5	Ngoc et al. [22]	2019	Vietnam	Adults living in the herbicide-sprayed and non-sprayed areas	Residential areas (cases and controls)	Prevalence of enamel dysplasia.

**Table 3:** Effects of dioxins on bones in animals.

No.	Author	Publication Year	Experimental Animals	Exposure Time	Exposure Dose	Health Effects (Outcomes)
Exposure during fetal period and/or lactation						
1	Miettinen et al. [23]	2005	Rat strains with different susceptibility	GD11, GD13, GD19, PND0, PND2, PND4	0.03, 0.1, 0.3, and 1 μg/kg of single oral doses to dams	Decreased bone length, cortical cross-sectional area, and bone density of tibia, femur, and femoral neck.
2	Nishimura et al. [24]	2009	C57BL/6J mice	day 1 after delivery	15 μg/kg of a single oral dose to dams	Abnormal calcification of the tibia, increased 1,25-dihydroxy vitamin D3, and decreased osteogenic biomarker activity. No alteration of bone resorptive marker activity.
3	Finnilä et al. [25]	2010	Female Sprague Dawley rats	GD 11	1 μg/kg of a single dose in gavage	Normal bone development, such as decreased plasticity, increased dynamic hardness, storage modulus, and composite modulus.
4	Yamada et al. [26]	2014	Pregnant ICR strain mice	Embryonic Day 12.5	40 μg/kg of a single dose in gavage	Palatal osteogenesis and myogenesis related with occurrence of cleft palates.
Exposure during adulthood						
1	Alaluusua et al. [12]	1993	Young adult male H/W rats	11–12 weeks of age	1000 μg/kg of a single intraperitoneal dose	Smaller skull size
2	Jamsa et al. [27]	2001	H/W and L/E rats	10–30 weeks of age	1.7 to 170 μg/kg of percutaneous doses	Decreased tibia size, 3-point bending fracture force, and stiffness of the tibia, but no alteration of bone mineral density (BMD).
3	Herlin et al. [28]	2010	Female L/E and H/W rats	10–30 weeks of age	0, 0.14, 1.4, 14 and 140 μg/kg for total dose of percutaneous doses	Altered bone geometry and bone biomechanical parameters, but no effect on bone mineral density parameters.
4	Herlin et al. [29]	2013	AhR knockout (Ahr(−/−)) and wild-type (Ahr(+/+)) mice	18–22 weeks of age	200 µg/kg for total dose in gavage	Harder bone matrix, thinner cortical bone, mechanically weaker bone, and increased trabecular bone volume fraction.
5	Fader et al. [30]	2018	Male and female C57BL/6 mice	PND25-53 (every 4 days)	0.01–30 μg/kg of oral doses	Increased trabecular bone volume of femur bone, inhibition of bone resorption markers, increased number of osteoblasts on the trabecular bone surface, increased regulator of osteoblast differentiation and mineralization, increased serum 1,25-dihydroxy Vitamin D3.

GD: Gestation day; PND: Postnatal day; H/W: Han/Wistar; L/E: Long/Evans.

**Table 4:** Effects of dioxins on bones in humans.

No.	Author	Publication Year	Country/Region	Target Population (Gender, Age, Residents/Patients)	Exposure Indicators	Bone Effect Markers
1	Akamine et al. [31]	1985	Japan	Yusho patients aged at 20 s to 60 s	dl-PCB and PCDF congeners in sera	Higher prevalence of sever alveolar bone resorption in patients compared with healthy subjects in the same age groups.
2	Shimizu et al. [32]	1992	Japan	Yusho patients	dl-PCB and PCDF congeners in sera	Increased morbidity of periodontal diseases with horizontal alveolar bone resorption during 12-year observation
3	Hodgson et al. [33]	2008	Sweden	Residents aged at 60–81 year (154 men and 167 women) living in Baltic Sea coast areas	Five dl-PCBs PCB118, three non-dl PCBs, and p,p′-DDE in blood	Low bone density correlated with PCB118 in men, but positive correlation between bone density and PCB118 in women.
4	Eskenazi et al. [7]	2014	Seveso, Italy	350 women who were under 20 years old in 1976	TCDD in sera taken immediately after the explosion	Bone density and size and strength indices of the three hip-forming bone regions. Better bone structure in highly exposed women, but no association between bone density and TCDD.
5	Fukushi et al. [34]	2016	Japan	Residents (262 women and 227 men) including Yusho patients (61.5% in women and 69.6% in men)	PCDDs, PCDFs, and non-ortho PCBs in blood	1,2,3,4,6,7,8-HpCDD may have a negative effect on bone mineral density in women, but serum levels of this conjugate were not increased in patients with Yusho.

## Data Availability

Not applicable.
